# Who Was It?

**Published:** 1880-10

**Authors:** 


"WHO WAS IT?"—If our sister Sawtelle will refer to a formula for pneumonia, copied into the *Medico Literary Journal*, some three or four numbers back, she will see the name of the "professor" in connection with it.

*Mephitis Americana*, and not Me*m*phitis Americana, was the animal intended in the *second line*, at page 444, last issue. Printers' "devils," though an exceedingly valuable and useful set of fellows, often play—well, havoc with our intentions.

Wm. R. Warner & Co's agent has placed several samples of their valuable preparations on our desk, which seem fully up to the standard so long maintained by that trustworthy establishment.

